# Mesoscopic structural damage and permeability evolution of Shale subjected to freeze–thaw treatment

**DOI:** 10.1038/s41598-022-06263-y

**Published:** 2022-02-09

**Authors:** Jun-Guang Wang, Zhang-Qing Xuan, Qiao Jin, Wei-Ji Sun, Bing Liang, Qing-Rong Yu

**Affiliations:** Liaoning Technical University, Fuxin, China

**Keywords:** Environmental sciences, Engineering

## Abstract

To study the mesoscopic damage and permeability evolution characteristics of rock under freeze–thaw (F–T) cycles, freeze–thaw cycle experiments were carried out of shale under different F–T temperatures and numbers of cycles, and nuclear magnetic resonance (NMR) and permeability experiments of shale were conducted thereafter. On the basis of these experiments, the pores and permeability of the F–T shale were analyzed, and the existing permeability model is modified and improved; Therefore, the mesoscopic damage evolution characteristics and permeability evolution law of the F–T shale are obtained. It was found that with increasing number of cycles, the pore structure of the rock samples changed as the pore size expanded and the number of pores increased, and the average porosity also increased correspondingly. The influence of the F–T cycle temperature on the shale permeability was not as notable as that of the number of F–T cycles. Based on the SDR-REV permeability model, the spectral area ratio parameters of large pores and fractures in the *T*_*2*_ spectrum were considered for correction, and a direct relationship between the permeability, F–T temperature and number of cycles was obtained via regression analysis. Compared to the experimental results, it was found that the modified model achieved a good applicability. The damage and permeability characteristics of shale under different F–T conditions were analysed from a microscopic perspective, which could yield an important reference for engineering construction in frozen soil areas.

## Introduction

Under the influence of temperature, rivers, snow and ice in cold regions, many geotechnical engineering projects inevitably face a certain period of F–T cycles. From the development of resources to the construction of subways and mining engineering, the influence of frozen rock on engineering safety should be considered. Therefore, experimental studies on the evolution of mesoscopic rock damage under different freezing conditions are of great significance for the development and optimization of engineering in frozen regions^1,2^. Hence, the effect of F–T cycles on shale, a widely distributed rock, cannot be neglected.

Many scholars have conducted much research on rock freezing and thawing. The F–T process is a physical process that exerts a notable influence on the rock strength^3^. Cui Jiangfeng and Si Guangyao^4^ applied the finite difference method in steady-state numerical simulations and characterized the effect of microfractures on the shale seepage capacity. Hao Li, Siddharth Misra, and Jiabo He^5^ developed two machine learning techniques based on neural networks and generated NMR *T*_*2*_ distributions in the absence of hard-to-obtain NMR *T*_*2*_ distribution logs. Hiroaki Izumiuama^6–8^ et al. studied the influence of freeze–thaw cycles on the porosity of granite, sandstone and other rocks and established a damage extension model for different weathered bedrock types. Coralie Genty and Jerry L. Jensen^9^ et al. greatly improved the success rate of the identification of rock pore types with complex internal structures by splitting $T_{2}$ into three Gaussian components and classified rock pore types using Bayesian methods. Zhu^10^ conducted a digital test of uniaxial compression deformation damage of marble samples and analysed the evolution characteristics of microcracks. Li Jielin and Zhu Longyun^11^ studied the influence of pore water on sandstone pore changes under freeze–thaw cycles. Yao Yanbin and Liu^12^ applied NMR technology to shale research and proposed an accurate technique to characterize the shale porosity. Li Jielin^13–15^ et al. studied the influence of freeze–thaw cycles on sandstone dynamic properties and pore structure. Liu Hanwen^16^ established a calculation model of pore volume deformation for sandstone under freeze–thaw cycles and obtained the change law of the pore volume. Yu Jin and Zhang Xin^17^ investigated the influence degree of different sandstone pores under the action of water chemistry and freeze–thaw cycles. Zhu Linqi^18^ and Bai Songtao^19–21^ et al. established a new model to obtain the permeability in different ways. Zhai Cheng and Sun Yong^22^ studied the evolution of coal porosity and permeability with the number of circulating freeze–thaw cycles. Qin Lei^23^ found that the SDR model highly agrees with the gas permeability. Jiangfeng Cui, Long Cheng, and Lei^24^ employed a 2D numerical model to explore the normalized equivalent permeability and representative elementary area (REA) of oil shale in detail incorporating the effects of kerogen.

The influence of the rock material under freeze–thaw cycles can be attributed to material deterioration caused by periodic or nonperiodic material loading^25–27^. Previous experimental studies on freeze–thaw cycles focused on the influence of freeze–thaw cycles on rock materials, while the impact of freezing temperature variation on rock materials is relatively limited. In addition, most studies of the influence of F–T conditions considered coal, sandstone and granite, and relatively few studies considered shale. Therefore, this paper carries out shale NMR and permeability experiments at different freezing temperatures and number of F–T cycles. Based on the pore structure changes and permeability of shale after the F–T process, we studied the mesoscopic damage and permeability evolution characteristics of shale under F–T cycle conditions to provide a reference for the F–T shale conditions encountered in engineering practice.

The innovation of this paper encompasses the consideration of the influence of different F–T temperatures and number of cycles on the shale permeability. Through experiments, we established a porosity and permeability evolution model considering both the F–T temperature and number of cycles, and we verified the accuracy of the model. The research results provide a theoretical basis for the prevention and control of F–T disasters in open-pit mines in cold regions.

## Shale sample preparation and test procedures

### Samples

**Figure 1:**
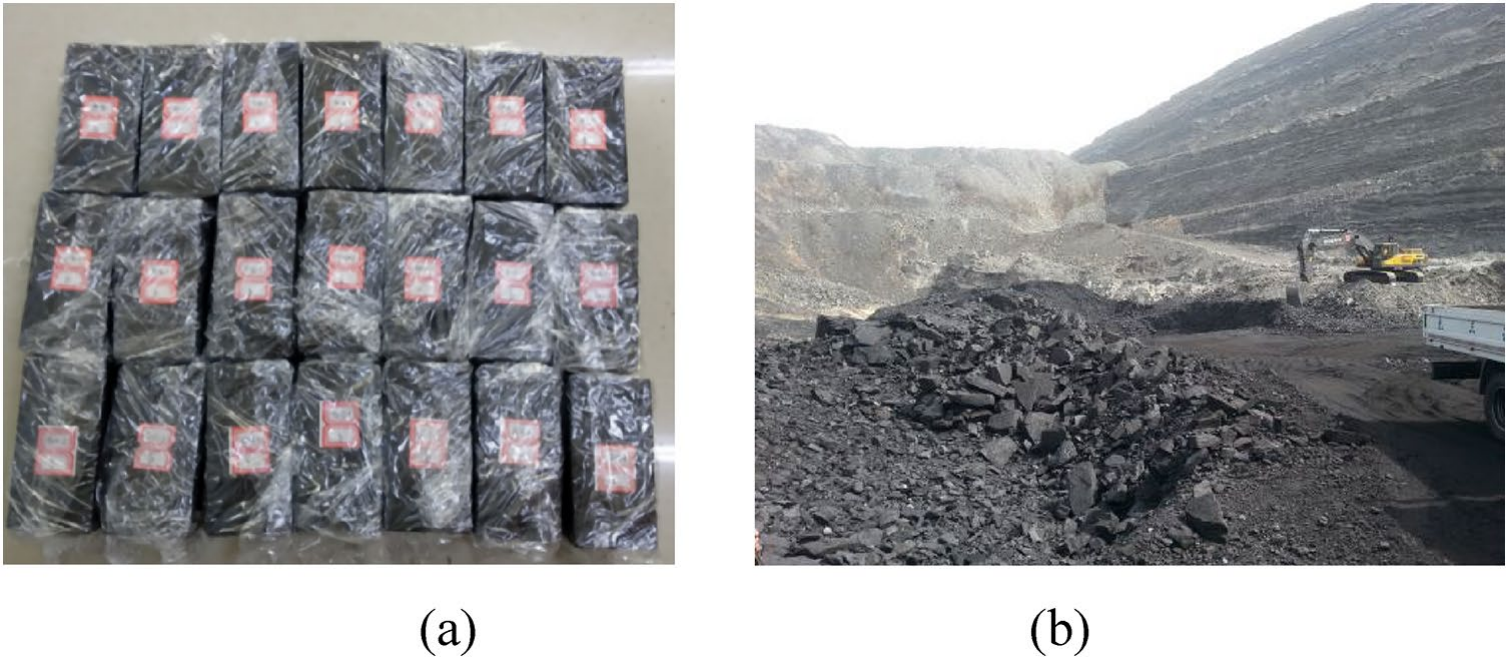
Select shale samples(left) and sampling the site(right).

Adopting the Haizhou open-pit mine in Fuxin, Liaoning, as the engineering background, shale rock blocks of Haizhou open-pit north nations with a good integrity were selected, and the rock blocks collected on site were sealed with plastic film. The outer surface of the shale samples is black, and there are no obvious cracks or fissures on the surface. The shale mineral composition in this area mainly includes quartz, carbonate minerals and clay minerals, among which there also occurs a small amount of pyrite, which easily expands upon contact with water. The moisture content in all shale samples is 1.2%. Then, each rock sample was processed into a 50 mm × 50 mm × 100 mm standard cube according to the experimental needs, and any rock sample test fragments that did not satisfy the above specification were removed^28^. Through ultrasonic testing of the specimens, the upper and lower surfaces of the specimens meeting the experimental requirements were polished with fine sandpaper. Select shale specimens after polishing are shown in Fig. 1.

### Experimental instrument

**Figure 2:**
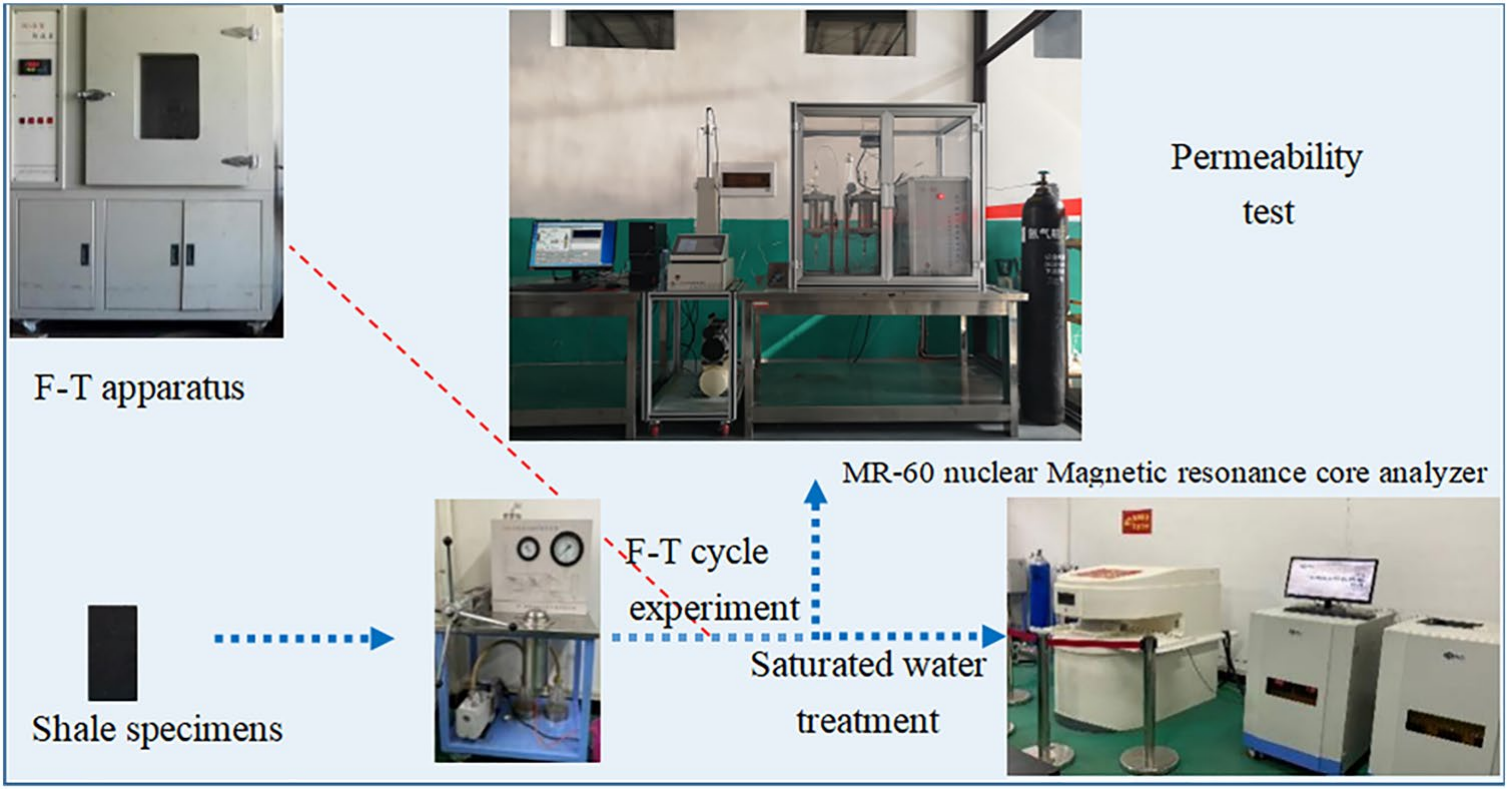
Overall experimental setup.

The main experimental instrument was an MR-60 nuclear magnetic resonance core analyser obtained from Suzhou Newmai Electronic Technology Co., Ltd. The main magnetic field strength is 0.51 T, the radio pulse frequency ranges from 1.0 ~ 49.9 MHz, and the radio frequency power is 300 W. Main test parameters: the main value of the RF signal frequency SF is 32 MHz, the magnet temperature T is 32 °C, the single-sampling point number TD is 1024, the cumulative sampling number NS is 32 times, the echo time TE is 0.233 ms, and the echo number NECH is 6000. The permeability experiment adopts an independently developed triaxial permeability instrument, mainly comprising a stress load system, temperature control system, rock sample holder system and data collection system. Instrument measurement precision: low permeability: ≤ 5%; medium and high permeability levels: ≤ 2%. The experimental equipment also included a freeze–thaw device (the lowest temperature of the freeze–thaw cycle experimental device could reach − 40 °C, and the highest constant-temperature precision of the cold source cycle device could reach ± 0.1 °C) and ultrasonic detector. The overall principle of the test device is shown in Fig. 2.

### Experimental principle.

The NMR *T*_*2*_ spectral distribution curve can represent the rock internal structural information, such as the pore size, pore number and pore ratio. The *T*_*2*_ value reflects the pore size and the larger the *T*_*2*_ value is, the larger the aperture size. The *T*_*2*_ spectral peak area is a fixed integral of the *T*_*2*_ spectral curve, the area size is proportional to the number of pores, and the area proportion of the different peaks reflects the proportion of different pore sizes in the rock mass^29,30^. In the permeability test, the pressure acting on the upper and lower specimen surfaces was controlled through the upstream and downstream inlet ends, respectively, of the pressure chamber. After specimen saturation, the osmotic pressure difference was obtained by considering the pressure at the upper and lower downstream inlets, and the computer system automatically recorded the change in pressure on the upper and lower surfaces of the rock samples over time and then calculated the permeability of the rock samples^31^.

### Test procedures

Before the experiment, the quality and geometric size of all specimens were determined via ultrasonication, and rock samples with relatively concentrated wave velocities and densities were selected as experimental rock samples. After the test, 2 rock samples were removed as the control, and the remaining 30 rock samples were divided into 2 groups. The first group of 15 rock samples was tested via NMR after F–T cycle experiments, and the second group of 15 rock samples was subjected to permeability tests after F–T cycle experiments. The F–T cycle experiments of the 30 rock samples were performed under different freezing conditions. According to the actual environmental conditions of the project, the freezing temperature was set to − 5 °C, − 15 °C and − 30 °C, and the specimens were maintained under constant-temperature conditions at 20 °C for 12 h after freezing for 12 h. This procedure constituted a freeze–thaw cycle experiment, and after the F–T cycle experiment, each rock sample was vacuumed and saturated with a vacuum water saturation device before the NMR experiment, and the instrument was calibrated with calibration samples. In addition, the calibration method employed the peak area method. A confining pressure of 5 MPa was applied during nuclear magnetic resonance measurement. Nuclear magnetic relaxation was measured in control rock shale samples in conjunction with the first set of shale specimens after the F–T experiments. NMR *T*_*2*_ curves of shale were obtained through inversion of the measurement results.

**Figure 3:**
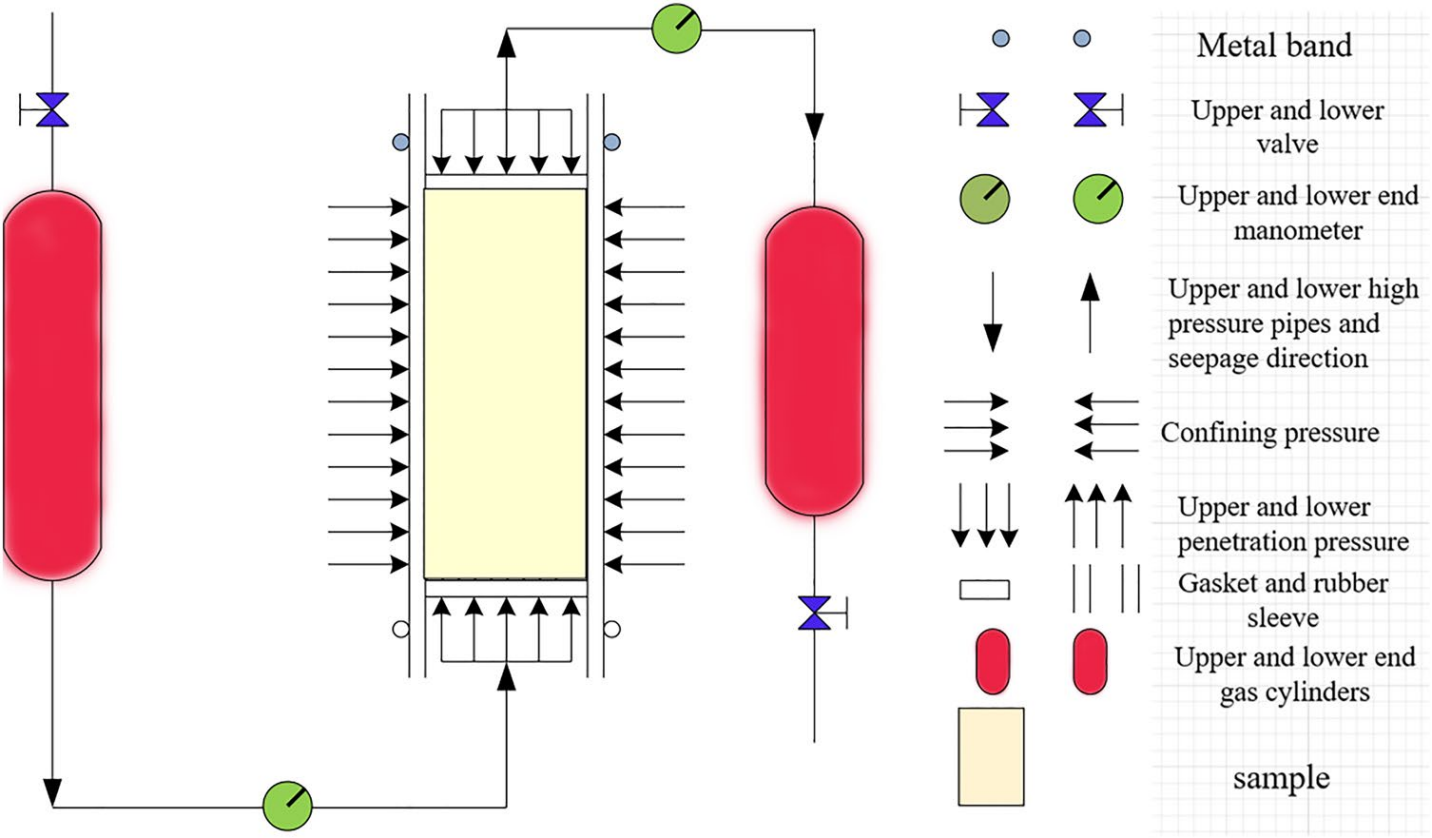
Principle of osmotic pressure experiment.

The principle of the osmotic pressure experiment is shown in Fig. 3. We evaluated the second set of 15 samples after the F–T cycle experiments in terms of the pulse gas permeability using helium as the permeation medium. According to the engineering geology, hydrogeology and ground stress in Haizhou district, the confining pressure was set to 5 MPa and the osmotic pressure was set to 2 MPa. The pressure on the rock sample surface was recorded by the system over time, and the permeability of the rock sample was thus calculated. The calculation basis of the gas permeability of the rock sample is:

1 $$K_{1} = \frac{{\gamma \mu LV_{1} V_{2} }}{{AP_{f} \left( {V_{1} + V_{2} } \right)}}$$

where $P_{1}$ and $P_{2}$ denote the sensor and actual measured pressure values, respectively, $\gamma$ is the attenuation coefficient, $\Delta P$ is the initial pressure difference, *t* is the time for the establishment of the upstream and downstream pressures, $\mu$ is the viscosity coefficient of nitrogen, *L* is the length of the specimen, *A* is the specimen area, *k* is the permeability, ${\text{V}}_{1}$ is the capacity of the upstream air cylinder of the infiltration system capacity, ${\text{V}}_{2}$ is the capacity of the downstream gas cylinder, $P_{f}$ is the equilibrium osmotic pressure of the system, and *K*_*1*_ is the experimentally measured permeability value.

## Results and discussion

According to the recorded NMR *T*_*2*_ spectrum, the shale mainly contained micropores and a few mesopores. At a freezing temperature of − 5 °C, there were no large pores or cracks throughout the whole F–T cycle. However, at a freezing temperature of − 15 °C and under 10 F–T cycles or at a freezing temperature of − 30 °C and under 5 F–T cycles, the pores expanded and macropore and crack structures were observed. The porosity and permeability results at the different freezing temperatures and number of F–T cycles revealed that with increasing number of F–T cycles and decreasing freezing temperature, the average porosity and permeability of shale increased, and the influence of the number of F–T cycles on the porosity and permeability was highly notable.

### ***T***_***2***_ NMR spectral analysis

**Figure 4:**
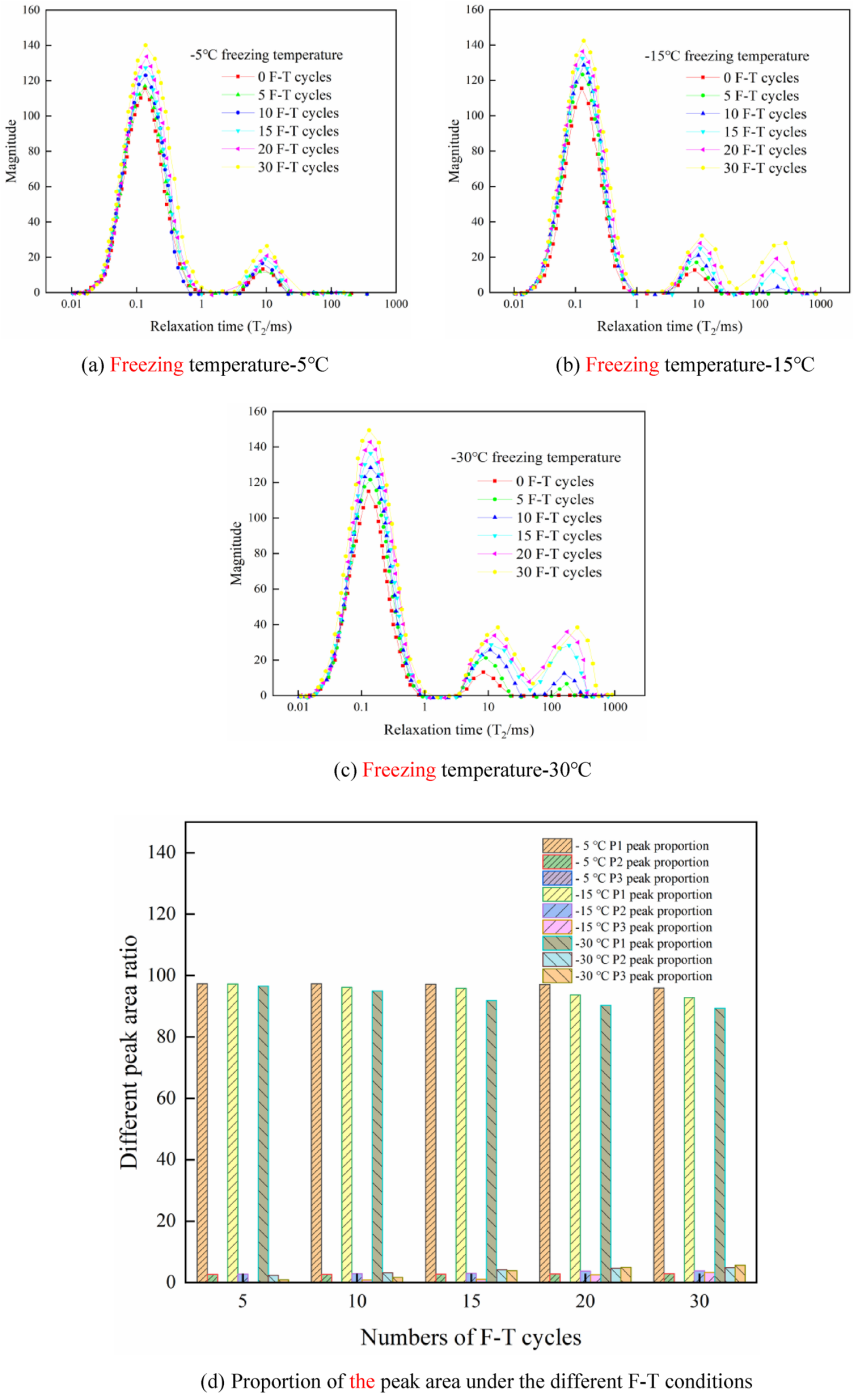
*T*_*2*_ distribution under the different F–T conditions.

The *T*_*2*_ spectral curves measured in the experiments were assessed to obtain the distribution of the *T*_*2*_ spectrum under the different numbers of F–T cycles at the three freezing temperatures, as shown in Fig. 4a–c. Figure 4d shows the proportion of each peak area under the different F–T conditions.

According to Fig. 4, at a freezing temperature of − 5 °C, the shale *T*_*2*_ spectrum is bimodal. Throughout the whole F–T cycle, the section where the *T*_*2*_ value is greater than 100 ms remains unchanged, and the proportion of the peak area does not change much between the various shale specimens. At this freezing temperature, the rock deterioration due to the F–T process is very limited. When the number of F–T cycles reaches 10, the curve section where the *T*_*2*_ value is greater than 100 ms gradually rises. The *T*_*2*_ curve gradually exhibits a trimodal morphology. At this time, large pores and crack structures are observed. At a freezing temperature of − 30 °C, according to the change characteristics of the *T*_*2*_ spectrum, the freeze expansion force generated during the F–T cycle experiments greatly increases the damage effect on the shale samples. Specifically, when the number of F–T cycles reached 5, the NMR *T*_*2*_ spectra of the shale samples exhibit a trimodal morphology. The peak increase with the number of F–T cycles is more pronounced. Moreover, the right peak increases the most notably from 10 to 15 cycles. Overall, the curve slightly shifts to the right from 20 to 30 cycles, but the increase decelerates. Based on the flatness of the curve, the three peak junctions occur sooner and become smoother after the F–T cycles, indicating a higher connectivity between the pores of the different sizes in the shale rock samples after the freeze–thaw cycles. Figure 4d reveals that the shale NMR *T*_*2*_ spectrum exhibits a typical bimodal structure, and the proportion of the P_1_ peak area is much higher than that of the P_2_ peak area, which is consistent with the initial pore distribution in shale, namely, micropores dominate, with a few mesopores and no large pores or fractures. At a freezing temperature of − 15 °C, the proportion of the P_1_ peak area gradually decreases, indicating that some micropores develop into mesopores and macropores. At a freezing temperature of − 30 °C, the proportion of the P_2_ and P_3_ peak areas greatly increases during the whole process, and the P_3_ peak area changes the most notably. Moreover, there occurs a small segment with a signal amplitude of 0 at the junction of these two peaks, indicating that the pore connectivity in the shale samples is poor.

### Variations in pore structure

According to international porosity classification standards^32^, the pore structure was divided into micropores (${\text{d}} < 100nm$, *T*_*2*_ > 2.5 ms), medium pore structures (${\text{100nm}} < {\text{d}} < 1000nm$, 2.5 ms < *T*_*2*_ < 100 ms) and large pores and crack structures (${\text{d}} > 1000nm$, *T*_*2*_ > 100 ms) based on the range of the pore sizes and corresponding *T*_*2*_ values.

To further study the variation in the pores of different sizes with the number of cycles at the various freezing temperatures, we determined the NMR *T*_*2*_ spectral area ratio before and after the F–T cycle experiments as follows:

2 $$S_{i} = \frac{{A_{{i_{n} }} }}{{A_{{i_{0} }} }}\;\left( {i = a,b,c,d} \right)$$

In the above equation, $i = a$ denotes the micropores and small pores, $i = {\text{b}}$ denotes the medium pores, $i = {\text{c}}$ denotes the large pores and crack structures, and $i = {\text{d}}$ denotes the total porosity, where $A_{{{\text{i}}_{{0}} }}$ is the *T*_*2*_ spectral area of the aperture corresponding to the shale samples not subjected to freeze–thaw cycles, and $A_{{{\text{i}}_{{\text{n}}} }}$ is the *T*_*2*_ spectral area of the corresponding aperture under the different freezing conditions. Since the different freeze–thaw cycle stages of the pore structures correspond to $T_{2} > 100ms$ under the various freezing conditions, the spectral area corresponding to $T_{2} > 100ms$ is first denoted as $A_{{{\text{c}}_{{0}} }}$.

**Figure 5:**
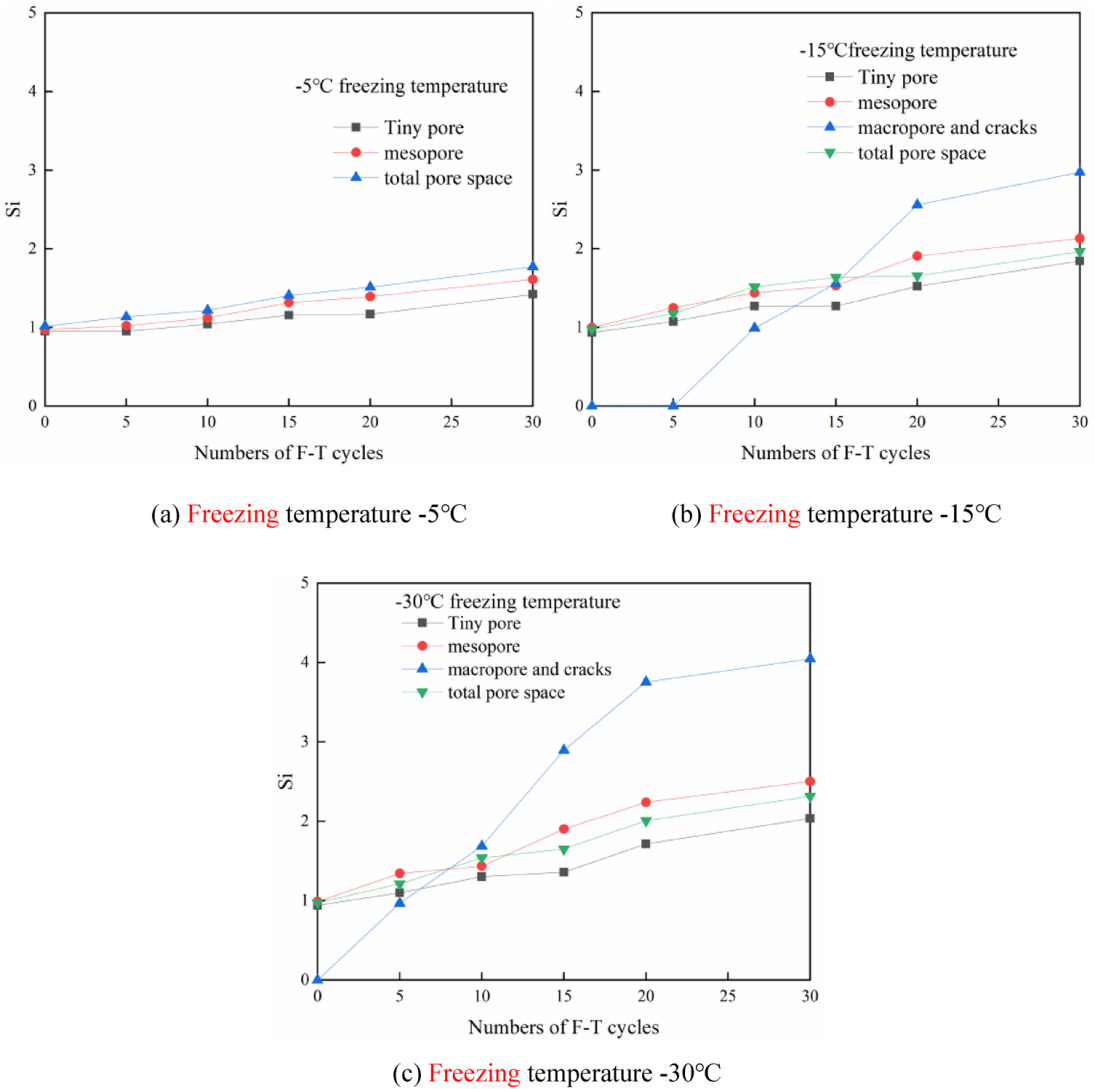
Variation in Si with the number of F–T cycles at different freezing temperatures.

Figures 5a–c show the results of *S*_*i*_ at the different freezing temperatures to illustrate the distribution rules of the pores of the different sizes. At a freezing temperature of − 5 °C, *S*_*a-d*_ slightly increased with the number of F–T cycles, which suggests that the proportions of micropores, small pores and medium pores in the rock samples increased. The increase in medium pores was greater than that in micropores and small pores. The proportion of pores of the different sizes changed little before 10 cycles, which indicates that micropores still occupied most of the entire pore structure. At a freezing temperature of − 15 °C, *S*_*c*_ was attained after 10 cycles, and *S*_*c*_ increased greatly from 10 to 20 F–T cycles, which demonstrates that large pores and cracks notably developed at this stage, while the increase in micropores was limited. The shale pore structure at this stage changed as small pores developed into medium and large pores. With increasing number of small pores, from 20 to 30 F–T cycles, *S*_*c*_ growth declined, and the development speed of the large pores and cracks was reduced. At a freezing temperature of − 30 °C, the most significant change in *S*_*c*_ occurred from 5 to 20 cycles. The *S*_*a-c*_ changes before 15 cycles occurred relatively slowly, which indicates that the number of pores and cracks increased due to freezing and thawing, while the development of micropores, small pores and medium pores decelerated. The *S*_*a-c*_ change degree increased beyond 15 cycles, small and medium pores were continuously generated in the rock samples, and the degree of *S*_*c*_ variation declined after 20 cycles. Large pores and cracks were more strongly affected by freezing and thawing than were micropores, small pores and medium pores, and the corresponding influence stage also occurred earlier than that of micropores, small pores and medium pores. We found that in regard to small pores, because the initial shale internal pore crack network was not well developed and there occurred less water, the expansion effect of water ice phase change was not obvious at the beginning. With increasing circulation, small pores were developed to a certain extent, the degree of water entering the pores gradually increased, and the expansion effect of water ice phase change gradually increased.

**Figure 6:**
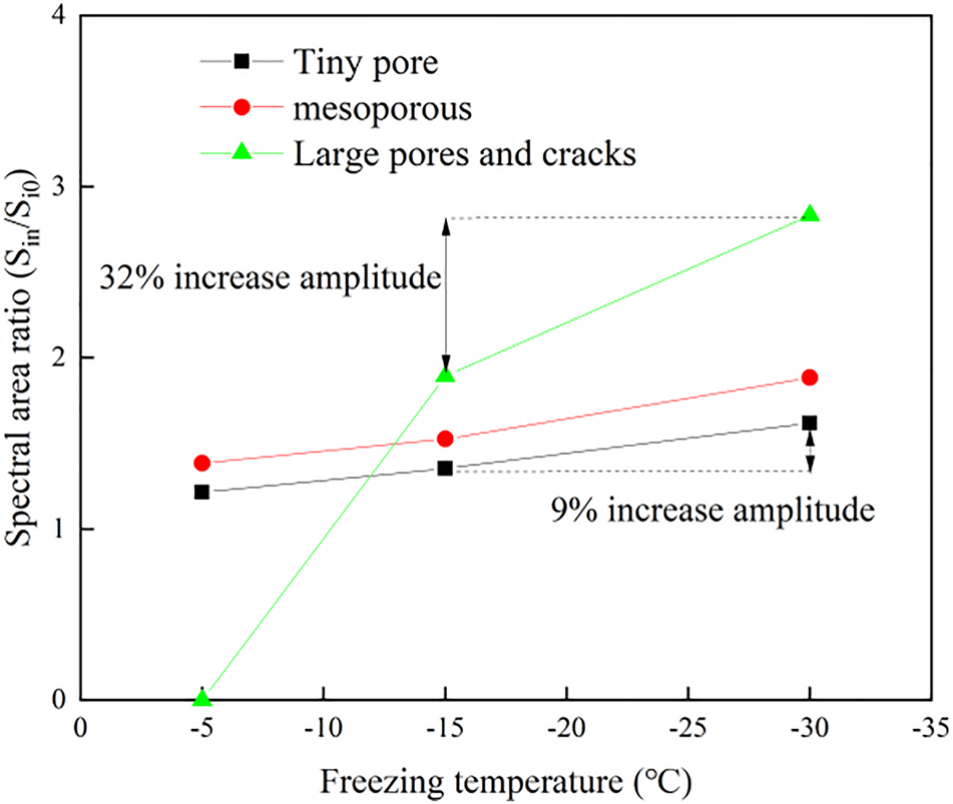
Proportion of the pore area after 15 cycles.

In the study of the shale pore diameter under the same number of cycles but different freezing temperatures, we determined the proportion of the pore spectral area after 15 F–T cycles. Figure 6 shows that after 15 F–T cycles, the pores of the different aperture sizes all increased with decreasing freezing temperature. Moreover, the proportion of the spectral area of the tiny pores and mesopores increased by approximately 9%, and with decreasing freezing temperature, the increase range expanded. However, the area proportion of the large pores increased by nearly 32% from − 15 to − 30 °C, and this increase indicates that the large pores and cracks were greatly affected by the freezing temperature.

### Analysis of the porosity and permeability

**Figure 7:**
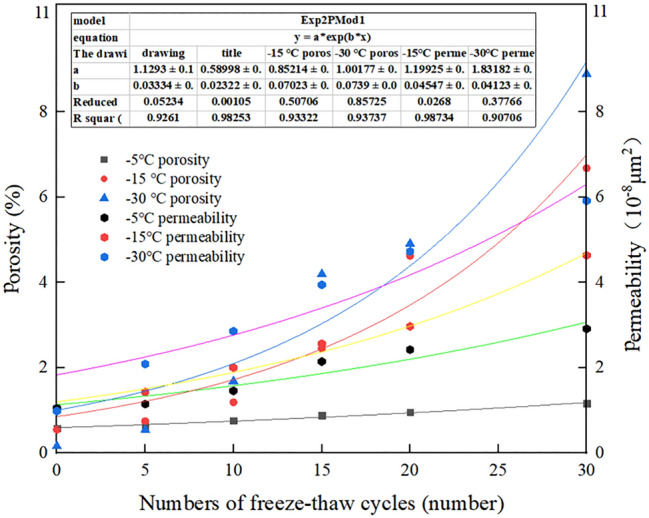
Evolution law of the shale permeability and porosity with the F–T cycles.

Figure 7 shows the changes in the nuclear magnetic resonance-based porosity and permeability measured in the shale experiments at the different freezing temperatures with increasing number of F–T cycles. With increasing number of F–T cycles and decreasing F–T cycle temperature, the average shale porosity increased. Through pore size analysis, the spectral area proportion of the pores with the different pore sizes increased with decreasing temperature, indicating that the number of pores of the different pore sizes increased, and the porosity also increased. Moreover, in the cyclic F–T process, the continuous expansion of micropores into medium- and large-sized pores resulted in an increasing shale porosity^20^. Therefore, the change in porosity was attributed to the pore size expanded and the number of pores increased.

As shown in Fig. 7, at a freezing temperature of − 5 °C, there occurred a small increase in rock porosity with the number of F–T cycles with no notable change in the shale permeability. When the number of F–T cycles reached 30, the shale permeability was 2.51 × 10^–8^ μm^2^, and the F–T process-induced permeability reached 0.61 × 10^–8^ μm^2^, which occurred at the same order of magnitude. According to the NMR results depicted in Fig. 4a, large pores and cracks were not observed throughout the whole F–T cycle, and the medium pores only changed slightly. The *T*_*2*_ geometric mean change range was limited. Therefore, the slight change in porosity did not notably improve the shale permeability. At a freezing temperature of − 15 °C, which reveals a more obvious change in permeability than that at a freezing temperature of − 5 °C. The permeability increased from the initial value of 0.61 × 10^–8^ μm^2^ to the final value of 6.71 × 10^–8^ μm^2^, i.e., the permeability increased by an order of magnitude. The permeability increased most notably from 15–30 F–T cycles, which coincided with an obvious increase in shale porosity. At a freezing temperature of − 30 °C, Fig. 7 shows the most significant increase with the number of F–T cycles, and after 30 cycles, the rock permeability reached 35.63 × 10^–8^ μm^2^. An increase in the initial permeability of one order of magnitude occurred. The notable increase attained in medium- and large-sized pores from 10–30 F–T cycles indicates that the development of large pores and fissure structures imposed a great impact on the permeability, and the permeability significantly increased with the porosity.

### Theoretical model

Based on the definition of the effective compression coefficient of rock^33^, we define the temperature-dependent porosity parameter $\alpha$, i.e., the change in porosity per unit change in temperature:

3 $$\alpha = \frac{1}{\phi }\frac{{{\text{d}}\phi }}{dT}$$

where T is the temperature and $\mathrm{\varphi }$ is the porosity. The result of the integrating of Eq. (3) is as follows:

4 $$\phi = \phi_{0} {\text{e}}^{{\alpha {\text{(T - T}}_{{0}} {)}}}$$

where $T_{0}$ is the initial temperature and $\phi_{0}$ is the porosity corresponding to temperature $T_{0}$. Figure 7 shows that the relationship between the porosity and number of F–T cycles adheres to an exponential function, as follows:

5 $$\phi = A{\text{e}}^{{{\text{Bx}}}}$$

where *x* is the number of F–T cycles and *A* and *B* are fitting coefficients. Hence, the porosity can be considered as:

6 $$\phi = C{\text{e}}^{{B\alpha {\text{(T - T}}_{{0}} {\text{)x}}}}$$

where *C* is a coefficient related to the initial porosity. The permeability is a measure of how easily fluid flows through rock pores and throats^34^. Ye Zhaohui^35^ demonstrated through experiments that the SDR-REV permeability model achieves a good applicability in the calculation of the permeability of low-permeability rocks. The SDR-REV permeability model can be expressed as:

7 $$K = D \times \phi^{E} \times T_{2g}^{c}$$

where *K* is the theoretically calculated permeability, D, E, and c are model parameters and *T*_*2g*_ is the geometric mean. The above model indicates that the influence of large pores and cracks on the permeability is much more notable than that of other pores. According to the above explanation and analysis of *Sc*, we introduced this parameter on the basis of Eq. (7):

8 $$K = D \cdot \phi^{E} \cdot S_{c} \cdot T_{2g}^{c}$$

Substituting the porosity expression (Eq. (6)) into Eq. (8) yields the following:

9 $$K = {\text{a}} \cdot {\text{e}}^{{b\alpha (T - T_{0} )x}} \cdot S_{c} \cdot T_{2g}^{c}$$

where $${\text{a}} = D \cdot C^{E}$$ and $${\text{b}} = B \cdot E$$.

**Figure 8:**
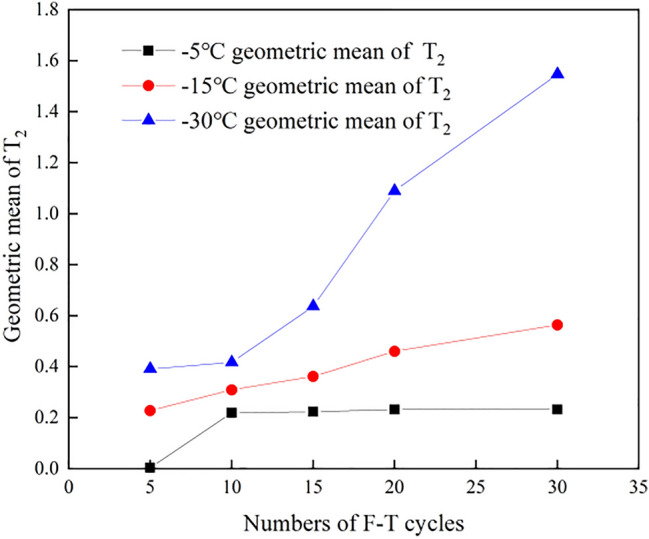
Geometric average over the cycles at the different freezing temperatures.

After regression analysis of the experimental permeability data listed in Table 1 and Fig. 8, parameters *a* = 2.62 × 10^–7^, *b* = 1.642 and *c* = 1.161 were obtained, and the correlation coefficient reached 0.98. Thus, Eq. (9) can be rewritten as follows:

10 $$K = 2.62 \times 10^{ - 7} \cdot {\text{e}}^{1.642\alpha (T - 25)x} \cdot S_{c} \cdot T_{{2{\text{g}}}}^{1.161}$$

**Table 1:** Experimental measurement of the permeability under the different F–T cycle conditions.

Number of F–T cycles	− 5 °C permeability (${10}^{{ - 8}} \mu m^{2}$)	− 15 °C permeability (${10}^{{ - 8}} \mu m^{2}$)	− 30 °C permeability (${10}^{{ - 8}} \mu m^{2}$)
0	1.39956	1.48722	1.18504
5	1.52486	1.85676	3.20107
15	1.86057	2.06849	8.52975
20	2.09398	3.19139	17.76631
25	2.40937	5.3249	20.96449
30	2.85505	7.02906	38.71187

**Figure 9:**
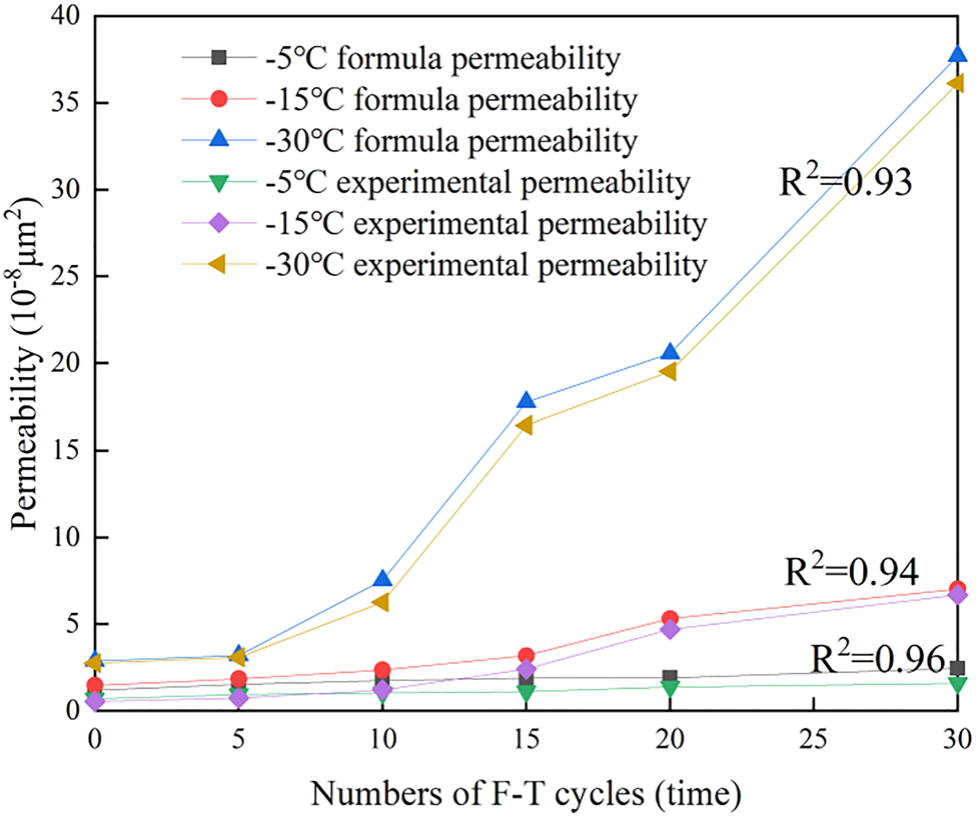
Comparison of the experimental and calculated permeability values.

To quantitatively analyse the impact of the freezing temperature and number of cycles on the shale permeability, the theoretical F–T shale permeability was calculated with Eq. (10), and the influence of the freezing temperature was not as notable as that of the number of cycles. The permeability values calculated with the model modified by *Sc* were compared to the experimental values, as shown in Fig. 9. The fitting correlation coefficient was higher than 0.93, indicating a good fit and verifying the applicability of the modified model.

## Conclusions

We analysed shale NMR spectra and pores under different F–T conditions, improved an existing permeability model through permeability experiments and quantitatively characterized the influence of the F–T temperature and number of cycles on the shale permeability. Based on An isotropic hypothesis of shale, and the following conclusions were obtained:

According to the NMR spectral data, the tested shale is mainly composed of micropores and a small number of mesopores. At the same freezing temperature, with increasing number of cycles, rock sample pore structure changes are mainly manifested by the micropores, small pores expand into medium pores, medium pores expand into large pores, and all pores of the various apertures increase. The effect of the number of F–T cycles on the large pores and cracks is more significant than that on the small pores and micropores. Under the same number of cycles, compared to the different freezing temperatures, the large pores and cracks are more notably affected by the temperature.

With increasing number of F–T cycles and decreasing freezing temperature, the average shale porosity increases at the three freezing temperatures, and the increase amount gradually rises. Via pore analysis considering the different pore sizes, it is found that the change in porosity is caused by the pore size expanded and the number of pores increased. At a freezing temperature of − 15 °C, the shale permeability increases significantly, while at a freezing temperature of − 30 °C, the permeability increases by one order of magnitude. Through comparison, it is found that the increases in shale porosity and permeability attain a good positive correlation. The influence of large pores and fractures on the shale permeability and porosity is more notable than that on the remaining pores. Moreover, compared to the F–T temperature, the number of F–T cycles exerts a more significant effect on the permeability and porosity.

In the analysis of the permeability model, based on the SDR-REV permeability model, *Sc* was considered in model modification. An improved shale permeability evolution model under F–T cycle treatment was obtained, which is directly related to the F–T temperature and number of cycles. The permeability calculation results were compared to the experimental results, indicating that the model achieves a good applicability. This permeability model can provide a reference for shale permeability calculations under similar working conditions.

## Supplementary Information

Supplementary Information.

## Data Availability

The data considered to support the findings of this study are included within the article.
