# Supplementary material for: Mesoscopic structural damage and permeability evolution of Shale subjected to freeze–thaw treatment
Source: Sci Rep. 2022 Feb 9;12:2202. doi: 10.1038/s41598-022-06263-y (PMC8828775; doi:10.1038/s41598-022-06263-y)
Supplement: Supplementary file 1 — Supplementary Information. [file 41598_2022_6263_MOESM1_ESM.docx]

During the experiment, we found cracks after freeze-thaw cycles, part of the figures is shown below:


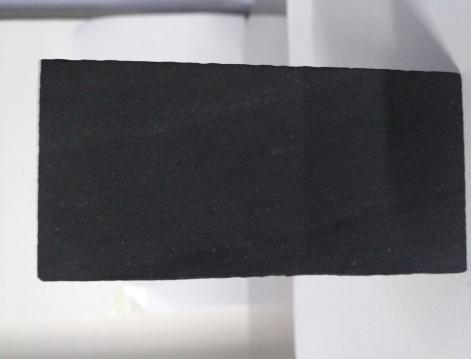

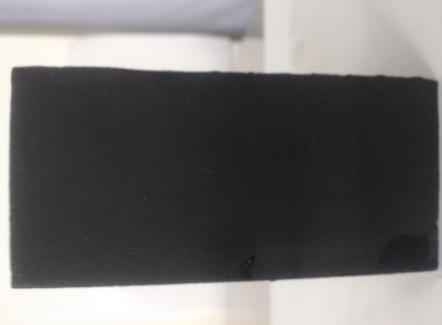

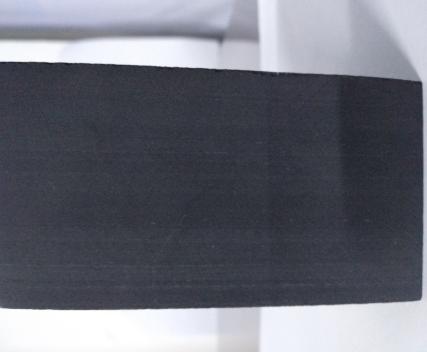


-5℃


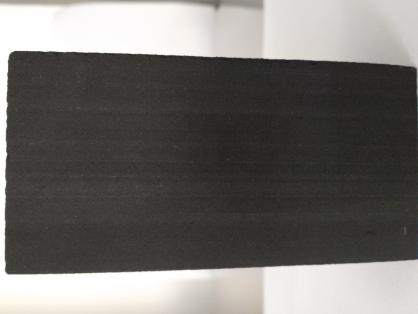

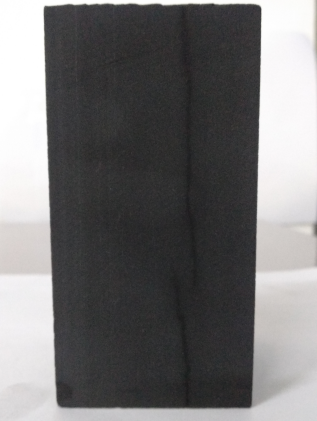

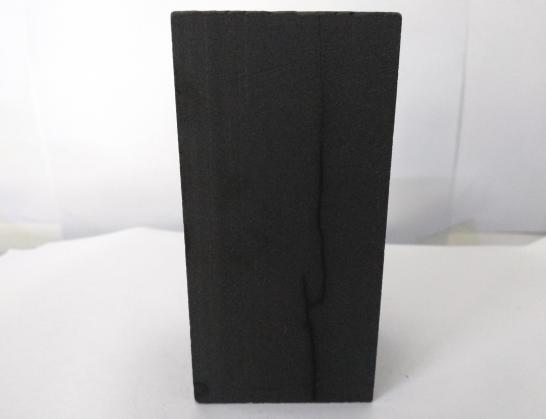


-15℃


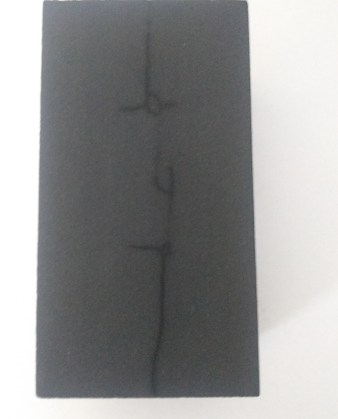

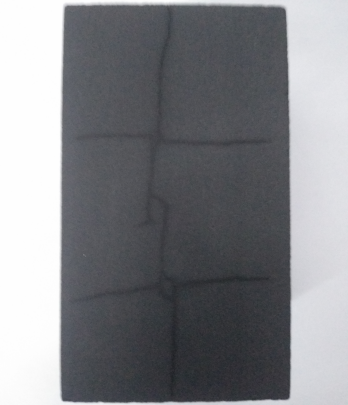

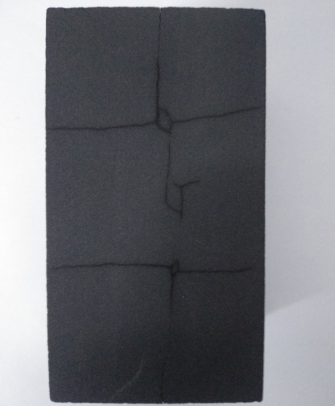


-30℃

Surface crack growth of test piece


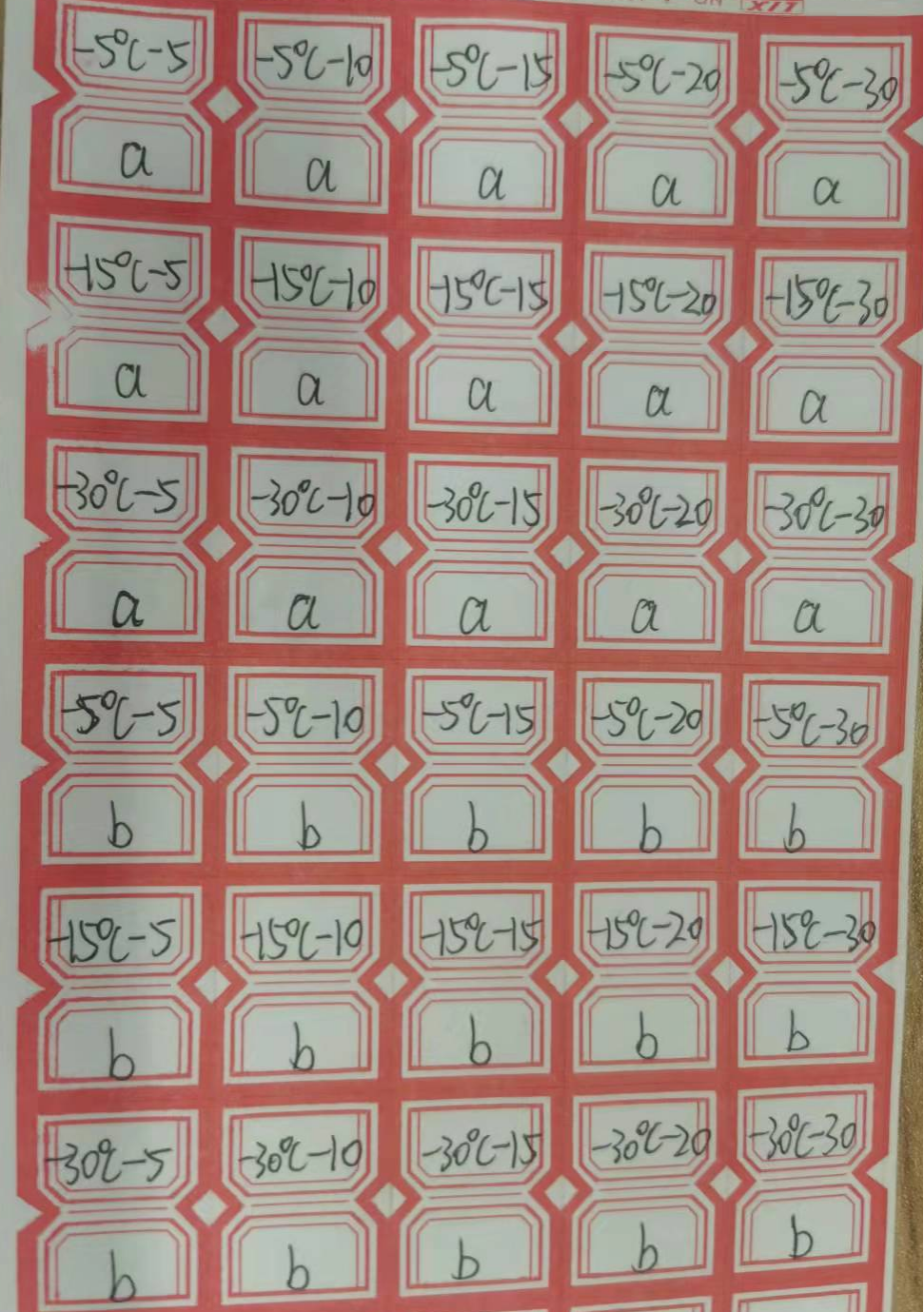


Supplementary figure 1
